# Quercetin as a Modulator of PTPN22 Phosphomonoesterase Activity: A Biochemical and Computational Evaluation

**DOI:** 10.3390/cimb46100662

**Published:** 2024-10-03

**Authors:** Abdulhakeem Olarewaju Sulyman, Tafa Ndagi Akanbi Yusuf, Jamiu Olaseni Aribisala, Kamaldeen Sanni Ibrahim, Emmanuel Oladipo Ajani, Abdulfatai Temitope Ajiboye, Saheed Sabiu, Karishma Singh

**Affiliations:** 1 Department of Biochemistry, Faculty of Pure and Applied Sciences, Kwara State University, Malete, Ilorin 241102, Nigeria; 2 Department of Nature Conservation, Faculty of Applied Sciences, Mangosuthu University of Technology, Durban 4031, South Africa; 3 Department of Biotechnology and Food Science, Faculty of Applied Sciences, Durban University of Technology, Durban 1334, South Africa; 4 Department of Chemistry and Industrial Chemistry, Faculty of Pure and Applied Sciences, Kwara State University, Malete, Ilorin 241102, Nigeria

**Keywords:** quercetin, cancer, PTPN22, in vitro and in silico studies

## Abstract

Cancer, a group of diseases characterized by uncontrollable cell proliferation and metastasis, remains a global health challenge. This study investigates quercetin, a natural compound found in many fruits and vegetables, for its potential to inhibit the phosphomonoesterase activity of protein tyrosine phosphatase nonreceptor type 22 (PTPN22), a key immune response regulator implicated in cancer and autoimmune diseases. We started by screening seven (7) natural compounds against the activities of PTPN22 in vitro. The initial screening identified quercetin with the highest percentage inhibition (81%) among the screened compounds when compared with ursolic acid that has 84%. After the identification of quercetin, we proceeded by investigating the effect of increasing concentrations of the compound on the activity of PTPN22. In vitro studies showed that quercetin inhibited PTPN22 with an IC_50_ of 29.59 μM, outperforming the reference standard ursolic acid, which had an IC_50_ of 37.19 μM. Kinetic studies indicated a non-competitive inhibition by quercetin with a Ki of 550 μM. In silico analysis supported these findings, showing quercetin’s better binding affinity (ΔGbind −24.56 kcal/mol) compared to ursolic acid, attributed to its higher reactivity and electron interaction capabilities at PTPN22′s binding pocket. Both quercetin and ursolic acid improved the structural stability of PTPN22 during simulations. These results suggest quercetin’s potential as an anticancer agent, meriting further research. However, in vivo studies and clinical trials are necessary to fully assess its efficacy and safety, and to better understand its mechanisms of action.

## 1. Introduction

Cancer, an encompassing term for a diverse group of diseases, can impact any part of the body. Characterized by the rapid proliferation of abnormal cells extending beyond their usual boundaries, cancerous cells have the potential to invade neighboring tissues and spread to distant organs, a process termed metastasis, which continues to be the predominant cause of mortality. This disease stands as a prominent global cause of mortality, affecting millions worldwide with the year 2020 which witnessed an estimated 10 million cancer-related deaths, a number projected to escalate due to unhealthy lifestyles, economic constraints in developing nations impeding effective treatment, and challenges linked to existing cancer therapies [1]. While the natural phenomenon of apoptosis facilitates cell replacement with more functional counterparts [2], cancerous cells evade programed cell death, accumulating and depriving surrounding cells of vital oxygen and nutrients. Consequently, these aberrant cells grow and form tumors, malignancies that manifest as irregular masses of tissue. Such tumors possess invasion capability against other tissues and propagate to distant sites, resulting in new tumors via metastasis [3].

Compounding the issue, cancerous cells disrupt immune function and induce irregular physiological changes. Lymph nodes, clusters of immune cells distributed throughout the body, can facilitate the spread of cancerous cells [4]. Leading cancer cases in 2020 include breast, lung, colon, rectum, prostate, skin, and stomach, with lung, colon, rectum, liver, and breast cancers being the primary causes of cancer-related deaths [5].

Treatment approaches encompass chemotherapy, radiotherapy, and surgery, each aiming to either cure or substantially prolong patients’ lives while enhancing their well-being. However, these methods have limitations. Chemotherapy, exemplified by drugs like cyclophosphamide and 5-fluorouracil, not only targets cancer cells but also healthy white blood cells, leading to compromised immunity and severe side effects such as appetite loss, headaches, diarrhea, vomiting, hair loss, fever, and low blood pressure. Radiotherapy’s focused radiation beam can damage nearby tissues, posing risks to organ function. Surgery, though capable of removing local tumors, struggles with addressing widespread cancers, potentially leaving behind untreated cells.

Epidemiological and cellular studies within the context of autoimmune diseases have illuminated the significance of protein tyrosine phosphatase nonreceptor type 22 (PTPN22) as a critical regulator of T cell receptor (TCR) signaling, pivotal in immune response modulation and cancer [6]. Given the function of protein tyrosine phosphatases (PTPs) like PTPN22 in tumorigenesis and disease, PTPN22’s involvement as a regulator of T cell activation, infection responses, autoimmunity, and anti-tumor immunity has emerged. Addressing current therapy limitations is essential, especially considering that certain cancer patients with the rs2476601 variant exhibited enhanced responses to checkpoint inhibitor immunotherapy, highlighting PTPN22’s relevance as a druggable systemic target for cancer treatment [6,7]. The rs2476601 single nucleotide polymorphism (SNP) is a well-studied genetic variant located in the PTPN22 gene, which encodes the protein tyrosine phosphatase non-receptor type 22 (PTPN22). This protein is involved in regulating immune responses by deactivating signaling pathways in T cells. A specific allele of rs2476601, often referred to as the R620W variant (arginine to tryptophan substitution at position 620), has been associated with several autoimmune diseases like type 1 diabetes, rheumatoid arthritis (RA), systemic lupus erythematosus (SLE), Graves’ disease, psoriasis and psoriatic arthritis, and multiple sclerosis (MS). Therefore, the prospect of selectively inhibiting protein-tyrosine phosphatases (PTPs), including PTPN22, emerges as a promising therapeutic avenue across various human ailments, notably cancer. To contribute to this evolving landscape, our study is directed at inhibiting PTPN22 using quercetin.

## 2. Materials and Methods

### 2.1. Chemicals and Reagents

*p*-nitrophenyl phosphate (*p*-NPP), Tris–HCl, tetraoxosulphate (VI) acids (H_2_SO_4_), boric acid sodium hydroxide (NaOH), chloroform, ethyl acetate, standard quercetin, 2HNQ, 5HNQ, selamectin, doramectin, escin, cedrol, and ursolic acid were obtained from Sigma-Aldrich Co (The Old Brickyard, New Rd, Gillingham SP8 4XT, United Kingdom). All other chemicals and reagents used were of analytical grade.

### 2.2. Cloning and Construction of Recombinant Plasmids

The cloning and assembly of the PTPN22 recombinant plasmid were carried out at the Biomedical Science Laboratory, located within the School of Pharmacy and Biomolecular Science at Liverpool John Moores University, Liverpool, United Kingdom. The construct sequence for the recombinant PTPN22 is given below: TGGCGAATGGGACGCGCCCTGTAGCGGCGCATTAAGCGCGGCGGGTGTGGTGGTTACGCGCAGCGTGACCGCTACACTTGCCAGCGCCCTAGCGCCCGCTCCTTTCGCTTTCTTCCCTTCCTTTCTCGCCACGTTCGCCGGCTTTCCCCGTCAAGCTCTAAATCGGGGGCTCCCTTTAGGGTTCCGATTTAGTGCTTTACGGCACCTCGACCCCAAAAAACTTGATTAGGGTGATGGTTCACGTAGTGGGCCATCGCCCTGATAGACGGTTTTTCGCCCTTTGACGTTGGAGTCCACGTTCTTTAATAGTGGACTCTTGTTCCAAACTGGAACAACACTCAACCCTATCTCGGTCTATTCTTTTGATTTATAAGGGATTTTGCCGATTTCGGCCTATTGGTTAAAAAATGAGCTGATTTAACAAAAATTTAACGCGAATTTTAACAAAATATTAACGTTTACAATTTCAGGTGGCACTTTTCGGGGAAATGTGCGCGGAACCCCTATTTGTTTATTTTTCTAAATACATTCAAATATGTATCCGCTCATGAATTAATTCTTAGAAAAACTCATCGAGCATCAAATGAAACTGCAATTTATTCATATCAGGATTATCAATACCATATTTTTGAAAAAGCCGTTTCTGTAATGAAGGAGAAAACTCACCGAGGCAGTTCCATAGGATGGCAAGATCCTGGTATCGGTCTGCGATTCCGACTCGTCCAACATCAATACAACCTATTAATTTCCCCTCGTCAAAAATAAGGTTATCAAGTGAGAAATCACCATGAGTGACGACTGAATCCGGTGAGAATGGCAAAAGTTTATGCATTTCTTTCCAGACTTGTTCAACAGGCCAGCCATTACGCTCGTCATCAAAATCACTCGCATCAACCAAACCGTTATTCATTCGTGATTGCGCCTGAGCGAGACGAAATACGCGATCGCTGTTAAAAGGACAATTACAAACAGGAATCGAATGCAACCGGCGCAGGAACACTGCCAGCGCATCAACAATATTTTCACCTGAATCAGGATATTCTTCTAATACCTGGAATGCTGTTTTCCCGGGGATCGCAGTGGTGAGTAACCATGCATCATCAGGAGTACGGATAAAATGCTTGATGGTCGGAAGAGGCATAAATTCCGTCAGCCAGTTTAGTCTGACCATCTCATCTGTAACATCATTGGCAACGCTACCTTTGCCATGTTTCAGAAACAACTCTGGCGCATCGGGCTTCCCATACAATCGATAGATTGTCGCACCTGATTGCCCGACATTATCGCGAGCCCATTTATACCCATATAAATCAGCATCCATGTTGGAATTTAATCGCGGCCTAGAGCAAGACGTTTCCCGTTGAATATGGCTCATAACACCCCTTGTATTACTGTTTATGTAAGCAGACAGTTTTATTGTTCATGACCAAAATCCCTTAACGTGAGTTTTCGTTCCACTGAGCGTCAGACCCCGTAGAAAAGATCAAAGGATCTTCTTGAGATCCTTTTTTTCTGCGCGTAATCTGCTGCTTGCAAACAAAAAAACCACCGCTACCAGCGGTGGTTTGTTTGCCGGATCAAGAGCTACCAACTCTTTTTCCGAAGGTAACTGGCTTCAGCAGAGCGCAGATACCAAATACTGTCCTTCTAGTGTAGCCGTAGTTAGGCCACCACTTCAAGAACTCTGTAGCACCGCCTACATACCTCGCTCTGCTAATCCTGTTACCAGTGGCTGCTGCCAGTGGCGATAAGTCGTGTCTTACCGGGTTGGACTCAAGACGATAGTTACCGGATAAGGCGCAGCGGTCGGGCTGAACGGGGGGTTCGTGCACACAGCCCAGCTTGGAGCGAACGACCTACACCGAACTGAGATACCTACAGCGTGAGCTATGAGAAAGCGCCACGCTTCCCGAAGGGAGAAAGGCGGACAGGTATCCGGTAAGCGGCAGGGTCGGAACAGGAGAGCGCACGAGGGAGCTTCCAGGGGGAAACGCCTGGTATCTTTATAGTCCTGTCGGGTTTCGCCACCTCTGACTTGAGCGTCGATTTTTGTGATGCTCGTCAGGGGGGCGGAGCCTATGGAAAAACGCCAGCAACGCGGCCTTTTTACGGTTCCTGGCCTTTTGCTGGCCTTTTGCTCACATGTTCTTTCCTGCGTTATCCCCTGATTCTGTGGATAACCGTATTACCGCCTTTGAGTGAGCTGATACCGCTCGCCGCAGCCGAACGACCGAGCGCAGCGAGTCAGTGAGCGAGGAAGCGGAAGAGCGCCTGATGCGGTATTTTCTCCTTACGCATCTGTGCGGTATTTCACACCGCATATATGGTGCACTCTCAGTACAATCTGCTCTGATGCCGCATAGTTAAGCCAGTATACACTCCGCTATCGCTACGTGACTGGGTCATGGCTGCGCCCCGACACCCGCCAACACCCGCTGACGCGCCCTGACGGGCTTGTCTGCTCCCGGCATCCGCTTACAGACAAGCTGTGACCGTCTCCGGGAGCTGCATGTGTCAGAGGTTTTCACCGTCATCACCGAAACGCGCGAGGCAGCTGCGGTAAAGCTCATCAGCGTGGTCGTGAAGCGATTCACAGATGTCTGCCTGTTCATCCGCGTCCAGCTCGTTGAGTTTCTCCAGAAGCGTTAATGTCTGGCTTCTGATAAAGCGGGCCATGTTAAGGGCGGTTTTTTCCTGTTTGGTCACTGATGCCTCCGTGTAAGGGGGATTTCTGTTCATGGGGGTAATGATACCGATGAAACGAGAGAGGATGCTCACGATACGGGTTACTGATGATGAACATGCCCGGTTACTGGAACGTTGTGAGGGTAAACAACTGGCGGTATGGATGCGGCGGGACCAGAGAAAAATCACTCAGGGTCAATGCCAGCGCTTCGTTAATACAGATGTAGGTGTTCCACAGGGTAGCCAGCAGCATCCTGCGATGCAGATCCGGAACATAATGGTGCAGGGCGCTGACTTCCGCGTTTCCAGACTTTACGAAACACGGAAACCGAAGACCATTCATGTTGTTGCTCAGGTCGCAGACGTTTTGCAGCAGCAGTCGCTTCACGTTCGCTCGCGTATCGGTGATTCATTCTGCTAACCAGTAAGGCAACCCCGCCAGCCTAGCCGGGTCCTCAACGACAGGAGCACGATCATGCGCACCCGTGGGGCCGCCATGCCGGCGATAATGGCCTGCTTCTCGCCGAAACGTTTGGTGGCGGGACCAGTGACGAAGGCTTGAGCGAGGGCGTGCAAGATTCCGAATACCGCAAGCGACAGGCCGATCATCGTCGCGCTCCAGCGAAAGCGGTCCTCGCCGAAAATGACCCAGAGCGCTGCCGGCACCTGTCCTACGAGTTGCATGATAAAGAAGACAGTCATAAGTGCGGCGACGATAGTCATGCCCCGCGCCCACCGGAAGGAGCTGACTGGGTTGAAGGCTCTCAAGGGCATCGGTCGAGATCCCGGTGCCTAATGAGTGAGCTAACTTACATTAATTGCGTTGCGCTCACTGCCCGCTTTCCAGTCGGGAAACCTGTCGTGCCAGCTGCATTAATGAATCGGCCAACGCGCGGGGAGAGGCGGTTTGCGTATTGGGCGCCAGGGTGGTTTTTCTTTTCACCAGTGAGACGGGCAACAGCTGATTGCCCTTCACCGCCTGGCCCTGAGAGAGTTGCAGCAAGCGGTCCACGCTGGTTTGCCCCAGCAGGCGAAAATCCTGTTTGATGGTGGTTAACGGCGGGATATAACATGAGCTGTCTTCGGTATCGTCGTATCCCACTACCGAGATATCCGCACCAACGCGCAGCCCGGACTCGGTAATGGCGCGCATTGCGCCCAGCGCCATCTGATCGTTGGCAACCAGCATCGCAGTGGGAACGATGCCCTCATTCAGCATTTGCATGGTTTGTTGAAAACCGGACATGGCACTCCAGTCGCCTTCCCGTTCCGCTATCGGCTGAATTTGATTGCGAGTGAGATATTTATGCCAGCCAGCCAGACGCAGACGCGCCGAGACAGAACTTAATGGGCCCGCTAACAGCGCGATTTGCTGGTGACCCAATGCGACCAGATGCTCCACGCCCAGTCGCGTACCGTCTTCATGGGAGAAAATAATACTGTTGATGGGTGTCTGGTCAGAGACATCAAGAAATAACGCCGGAACATTAGTGCAGGCAGCTTCCACAGCAATGGCATCCTGGTCATCCAGCGGATAGTTAATGATCAGCCCACTGACGCGTTGCGCGAGAAGATTGTGCACCGCCGCTTTACAGGCTTCGACGCCGCTTCGTTCTACCATCGACACCACCACGCTGGCACCCAGTTGATCGGCGCGAGATTTAATCGCCGCGACAATTTGCGACGGCGCGTGCAGGGCCAGACTGGAGGTGGCAACGCCAATCAGCAACGACTGTTTGCCCGCCAGTTGTTGTGCCACGCGGTTGGGAATGTAATTCAGCTCCGCCATCGCCGCTTCCACTTTTTCCCGCGTTTTCGCAGAAACGTGGCTGGCCTGGTTCACCACGCGGGAAACGGTCTGATAAGAGACACCGGCATACTCTGCGACATCGTATAACGTTACTGGTTTCACATTCACCACCCTGAATTGACTCTCTTCCGGGCGCTATCATGCCATACCGCGAAAGGTTTTGCGCCATTCGATGGTGTCCGGGATCTCGACGCTCTCCCTTATGCGACTCCTGCATTAGGAAGCAGCCCAGTAGTAGGTTGAGGCCGTTGAGCACCGCCGCCGCAAGGAATGGTGCATGCAAGGAGATGGCGCCCAACAGTCCCCCGGCCACGGGGCCTGCCACCATACCCACGCCGAAACAAGCGCTCATGAGCCCGAAGTGGCGAGCCCGATCTTCCCCATCGGTGATGTCGGCGATATAGGCGCCAGCAACCGCACCTGTGGCGCCGGTGATGCCGGCCACGATGCGTCCGGCGTAGAGGATCGAGATCTCGATCCCGCGAAATTAATACGACTCACTATAGGGGAATTGTGAGCGGATAACAATTCCCCTCTAGAAATAATTTTGTTTAACTTTAAGAAGGAGATATACCATGGGCAGCAGCCATCATCATCATCATCACAGCAGCGGCCTGGTGCCGCGCGGCAGCCATATGGATCAGCGCGAAATTCTGCAGAAATTTCTGGATGAAGCGCAGAGCAAAAAAATTACCAAAGAAGAATTTGCGAACGAATTTCTGAAACTGAAACGCCAGAGCACCAAATATAAAGCGGATAAAACCTATCCGACCACCGTGGCGGAAAAACCGAAAAACATTAAAAAAAACCGCTATAAAGATATTCTGCCGTATGATTATAGCCGCGTGGAACTGAGCCTGATTACCAGCGATGAAGATAGCAGCTATATTAACGCGAACTTTATTAAAGGCGTGTATGGCCCGAAAGCGTATATTGCGACCCAGGGCCCGCTGAGCACCACCCTGCTGGATTTTTGGCGCATGATTTGGGAATATAGCGTGCTGATTATTGTGATGGCGTGCATGGAATATGAAATGGGCAAAAAAAAATGCGAACGCTATTGGGCGGAACCGGGCGAAATGCAGCTGGAATTTGGCCCGTTTAGCGTGAGCTGCGAAGCGGAAAAACGCAAAAGCGATTATATTATTCGCACCCTGAAAGTGAAATTTAACAGCGAAACCCGCACCATTTATCAGTTTCATTATAAAAACTGGCCGGATCATGATGTGCCGAGCAGCATTGATCCGATTCTGGAACTGATTTGGGATGTGCGCTGCTATCAGGAAGATGATAGCGTGCCGATTTGCATTCATTGCAGCGCGGGCTGCGGCCGCACCGGCGTGATTTGCGCGATTGATTATACCTGGATGCTGCTGAAAGATGGCAGCCAGGCGAAACATTGCATTCCGGAAAAAAACCATACCCTGCAGGCGGATAGCTATAGCCCGAACCTGCCGAAAAGCACCACCAAAGCGGCGAAAATGATGAACCAGCAGCGCACCAAAATGGAAATTAAATAATAACTCGAGCACCACCACCACCACCACTGAGATCCGGCTGCTAACAAAGCCCGAAAGGAAGCTGAGTTGGCTGCTGCCACCGCTGAGCAATAACTAGCATAACCCCTTGGGGCCTCTAAACGGGTCTTGAGGGGTTTTTTGCTGAAAGGAGGAACTATATCCGGAT.

#### Expression and Purification of Recombinant PTPN22

The human PTPN22 catalytic domain open-reading frame (ORF), spanning amino acids 1 to 303, was synthesized by Integrated DNA Technologies, Inc., Coralville, IA, USA. Subsequently, this ORF, optimized for *Escherichia coli* expression, was inserted into the pD441b-HsPTPN22cd plasmid, generating a fusion protein (His6-sfGFP-SUMO-HsPTPN22cd). The N-terminal His-tag facilitates IMAC purification, sfGFP enhances folding and solubility, and SUMO allows ULP1 protease recognition for cleavage, liberating HsPTPN22cd from its N-terminal fusion partner. The introduction of the C129S mutation employed PCR with specific primers, leading to the creation of the pD441b-HsPTPN22cd-C129S plasmid. After separate introduction into *E. coli* BL21 (DE3), confirmation of ORF sequences via Eurofins Biotech, and initiation of protein expression in TB medium, the purification process involved IMAC binding, sonication, and HisPrep FF 16/10 column chromatography with an ÄKTA explorer FPLC system. Subsequent treatment with in-house expressed and purified His-tagged ULP1, followed by a second HisPrep FF 16/10 column step, resulted in the concentration, buffer exchange, and freezing of the non-tagged target protein for future use.

### 2.3. Determination of PTPN22 Activity

The procedure for measuring phosphate activity was adapted from Mascarello et al. [8] with a slight modification. In essence, 10 µL of 10 mM PTPN22 was introduced into a reaction mixture (100 µL) comprising 50 µL of 92 mM Tris-HCL and 20 µL of distilled water. Commencing the reaction involved adding 20 µL of *p*-nitrophenyl phosphate (PNPP), followed by an incubation period at 37 °C for 10 min. Termination of the reaction was achieved by adding 100 µL of 0.5 M sodium hydroxide (NaOH). Subsequently, the absorbance of the mixture was assessed at 405 nm using a Spectra Max M3 multimode microplate reader, Model 110.

### 2.4. Screening of Compounds

Seven compounds (7) sourced from Sigma-Aldrich, UK, were initially screened. These compounds were individually acquired and combined to create a small compound library. Since the compounds are water-insoluble, they were dissolved in 100% DMSO. The library comprised sesquiterpenoids, lactones, flavonoids, naphthoquinones, and triterpenoids. Each compound was provided with a specific molecular weight and was subsequently diluted to a final concentration of 50 µM to assess their inhibitory effect on PTPN22 activity. For the inhibition assay, 10 µL of each compound at a concentration of 50 µM was added to a final reaction volume of 100 µL, following the method outlined by Sulyman et al. [9]. In the control reactions, 10 µL of 100% DMSO was used instead of the compound solutions, resulting in a final concentration of 10% DMSO. The activity measured in the presence of 10% DMSO was normalized and considered as the uninhibited reaction. From this initial screening, only quercetin was identified as a potent inhibitor of PTPN22.

### 2.5. Determination of Inhibition of PTPN22 Activity by Quercetin

To determine the effectiveness of each of the quercetin identified from the initial screen, we investigated the effect of increasing concentrations of quercetin on the activity of PTPN22. The assessment of PTPN22 inhibition by quercetin was conducted according to the methodology outlined by Igunnu et al. [10]. In brief, an eppendorf tube was employed to transfer 50 µL of 92 mM Tris HCl (pH 7.5), followed by the addition of 10 µL of distilled water. Subsequently, 10 µL of 10 µM PTPN22 and 10 µL of varying concentrations (62.5, 125, 250, 500, 750, 1000, and 1250 µM) of the isolated quercetin were added to the mixture. The mixture was vortexed and spined down. To initiate the reaction, 20 µL of 25 mM *p*-NPP was added, and the mixture was incubated at 37 °C for a period of 10 min. The reaction was halted by adding 100 µL of 0.5 M NaOH. The absorbance of the solution was measured at 405 nm using a Spectra Max M3 multimode microplate reader, Model 110. For establishing a control, ursolic acid was used in place of the various concentrations of isolated quercetin. 

#### Determination of the Kinetic Parameter of PTPN22

The inhibition pattern and kinetic parameters of PTPN22 were determined in accordance with the procedure previously elucidated by Sulyman et al. [9]. In brief, 10 µL of 10 µM PTPN22 was introduced into a reaction mixture (100 µL) composed of 50 µL of 92 mM Tris HCl, 10 µL of distilled water, and 10 µL of quercetin at concentrations of 62.5 µM and 250 µM. Additionally, 20 µL of varying concentrations (10, 20, 40, 60, 80, 160, 200, 240, and 300 µM) of *p*-NPP was combined with the reaction mixture to initiate the enzymatic reaction. The reaction mixture was subjected to an incubation period of 10 min at 37 °C, followed by termination through the addition of 100 µL of 0.5 M NaOH. The absorbance of the solution was measured at 405 nm using a Spectra Max M3 multimode microplate reader, Model 110. To establish a control, 62.5 µM and 250 µM of isolated quercetin were substituted with 10 µL of DMSO.

### 2.6. Molecular Docking and Dynamic Simulation 

**Figure 1:**
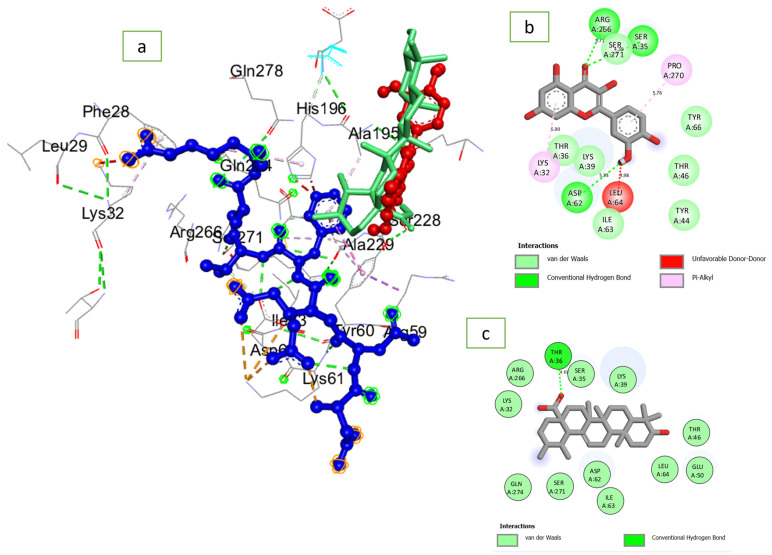
(**a**) Superimposition of the docked quercetin (red) and ursolic acid (green) at the native inhibitor (blue) binding pocket of PTPN22 (3BRH) showed relatively partial binding orientation; (**b**) 2D interaction plots of docked quercetin; and (**c**) ursolic acid at the binding pocket of PTPN22.

The molecular docking of quercetin against PTPN22 was done using Python Prescription (PyRx) v 0.9.5 [11] using ursolic acid as a reference standard. Briefly before docking, the 3D structure of quercetin and ursolic acid was obtained from PubChem (https://pubchem.ncbi.nlm.nih.gov/, accessed on 4 March 2024), while the co-crystallized structure of PTPN22 (3BRH) [12] was downloaded from the protein data bank (PDB) (https://www.rcsb.org, accessed on 4 March 2024). The optimization of the co-crystallized structure of PTPN22 was carried out using UCSF Chimera v 1.15 software via removal of water molecules and nonstandard amino acids. The ligands (quercetin and ursolic acid) on the other hand were optimized via the addition of Gasteiger charges using the Open Babel program present on PyRx v 0.9.5. Docking at the binding pocket of PTPN22 was done by selecting amino acid at the active site [12] with grid box coordinate corresponding to [center (x: −21.29; y: 29.94; z: 70.42), size (x: 20.22; y: 25.8; z: 25.0)]. Following molecular docking, the docking study was validated using the superimposition technique at the native inhibitor binding pocket of PTPN22 (3BRH). The root mean square deviation (RMSD) of the orientation of the docked compounds from the native inhibitor on 3BRH was relatively measured using Discovery Studio v21.1.0 [13] (Figure 1). For further molecular dynamic study, the orientation of the docked compounds with the highest docking scores was saved in PDB format.

The CHPC’s AMBER 18 package facilitated a 100 ns molecular dynamics (MDs) simulation of quercetin and ursolic acid against PTPN22. The AMBER force field’s FF18SB variant described the systems, and atomic partial charges for quercetin and ursolic acid were generated using ANTECHAMBER with the general amber force field (GAFF) and restrained electrostatic potential (RESP) approaches. Protonation states of PTPN22 were assigned using the AMBER LEaP module, considering amino acid residue correct protonation states. Systems were set in an orthorhombic box of TIP3P water molecules with residues numbered 1–296, ensuring proximity to box edges. Equilibration involved initial steps, energy minimization, heating, and system equilibration, maintaining specific temperature and pressure. Hydrogen bond restrictions using SHAKE, randomized seeding, and a 2 fs step size were employed in simulations (NPT ensemble). Langevin thermostat and pressure-coupling maintained conditions, and post-dynamic data analysis included RMSD, RMSF, ROG, SASA, and ΔGbind determination using the MMGBSA technique from 100 ns MD trajectory snapshots [14,15].

ΔGbind = G_complex_ − (G_receptor_ + G_ligand_) (1)

ΔGbind = −TS + (G_sol_ + E_gas_) (2)

E_ele +_ E_int_ + Evdw = E_gas_ (3)

−(G_GB_ − G_SA_) = G_sol_ (4)

γSASA = G_SA_ (5)

where Egas = the gas-phase energy, E_int_ = the internal energy, E_ele_ = coulomb energy, E_vdw_ = the van der Waals energy, Gsol = solvation-free energy from polar state, G_GB_ = solvation-free energy from polar state non-polar states, S = total entropy, and T = temperature.

The interaction plots of quercetin and ursolic acid against PTPN22 were visualized and analyzed with Discovery Studio v21.1.0 [13]. 

#### Quantum Chemical Calculations

This investigation aimed to predict the molecular characteristics of quercetin and ursolic acid using the widely adopted DFT/B3LYP/631G/+ (d, p) basis set [16,17]. The Gaussian 16 program package from the Center for High-Performance Computing was employed for analysis, and GaussView 6 software V. 6.0.16 was used for result evaluation. Conceptual density functional theory (CDFT) descriptors, including softness, hardness, electrophilicity index, energy gap, ionization energy, and chemical potential were then computed from the energies of the frontier lowest unoccupied molecular orbital (LUMO) and highest occupied molecular orbital (HOMO), considering Parr and Pearson’s interpretation of DFT [18] and Koopmans’ theorem on the correlation of ionization potential (*I*) and electron affinities (*E*) with HOMO and LUMO energy [19]. The calculations were performed using the following equations:

[E_LUMO − E_HOMO] (eV) = energy gap (Δ*E*) (6)

[*I* = −E_HOMO] (eV) = ionization energy (*I*) (7)

[ *A =* −E_LUMO) = electron affinity (*A*) (8)

[*η =* (*I* − *A*)/2] (eV) = hardness (*η*) (9)

[*S =* 1/*η*] (eV) = softness (*S*) (10)

[*χ* = (*I* + *A*)/2] (eV) = electronegativity (*χ*) (11)

[*μ =* −*χ =* −(*I* + *A*)/2] (eV) = chemical potential (*μ*) (12)

[*ω = μ*^2^/2 *η*] (eV) = electrophilicity index (*ω*) (13)

### 2.7. Statistical Analysis of Data

Unless indicated otherwise, all enzyme assays were conducted in triplicate, and the outcomes are presented as the mean ± standard error of the mean (SEM). The statistical analysis was executed through one-way analysis of variance (ANOVA) followed by the Duncan multiple range tests. Significance was considered at the 5% confidence level (*p* < 0.05). The software utilized for data analysis was GraphPad Prism 9.2.0 (GraphPad, La Jolla, CA, USA).

## 3. Results

### 3.1. Initial Screening of Compounds

**Figure 2:**
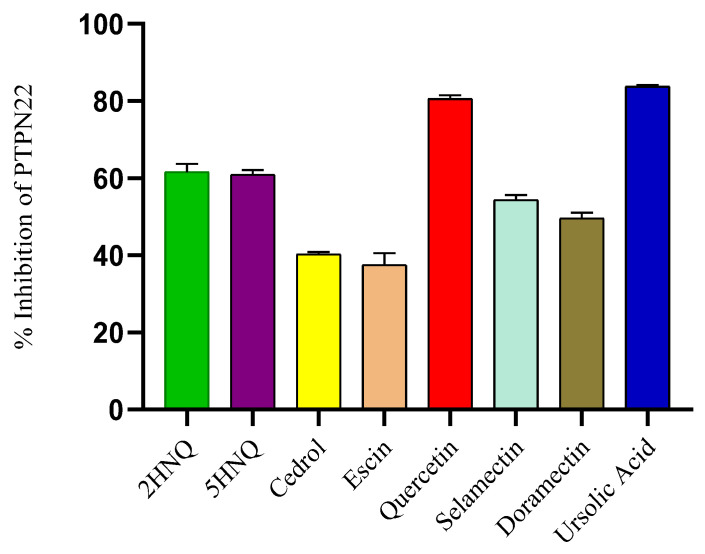
The screening of the small molecular weight compound is expressed as percentage inhibition. Values represent the mean ± standard error of the mean (SEM) from triplicate determinations, with statistical significance determined at a 5% confidence level (*p* < 0.05).

The results from the initial screening of the compounds at 50 µM, shown in Figure 2, indicate that the inhibitory potential against PTPN22 was in the order of quercetin (81%) > 2HNQ (62%) > 5HNQ (61%) > selamectin (54%) > doramectin (50%) > cedrol (40%) > escin (38%), compared to ursolic acid, which showed an 84% inhibition (Figure 2). From this initial screening, we identified quercetin as the only compound that competes favorably with ursolic acid, which was used as the reference inhibitor of PTPN22.

### 3.2. Inhibition of PTPN22 Activity by Quercetin

**Figure 3:**
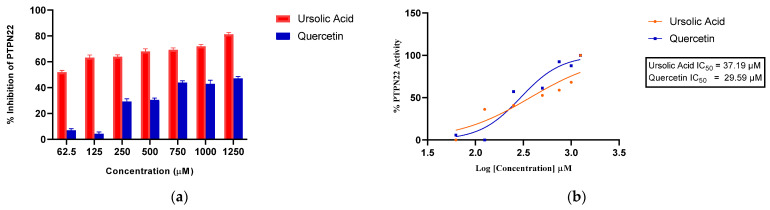
(**a**) Concentration dependent inhibition of quercetin and ursolic acid. (**b**) IC_50_ graph of quercetin and ursolic acid against PTPN22.

**Figure 4:**
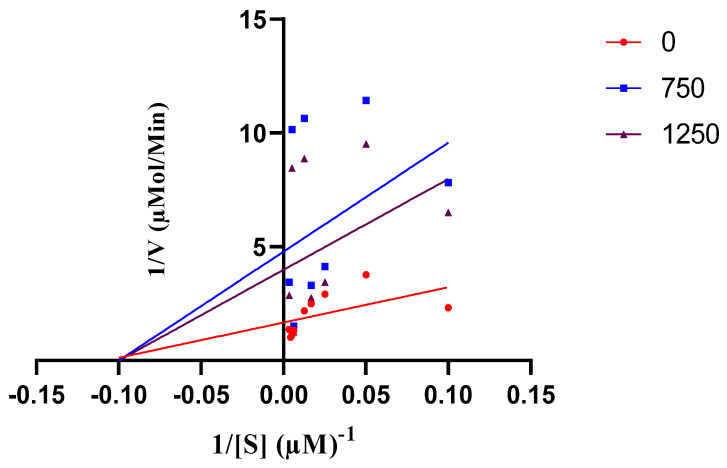
Lineweaver–Burk plot for the inhibition of PTPN22 by quercetin in the presence of different concentrations of quercetin (0, 750, and 1250 μM).

**Figure 5:**
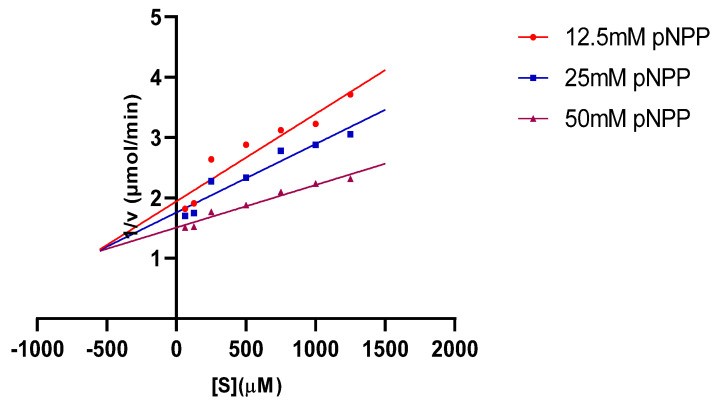
Dixon plots of protein tyrosine phosphatases non-receptor type 22 (PTP N22) inhibition by quercetin at various substrate (pNPP) concentrations (12.5, 25, and 50 mM).

The in vitro experiments demonstrated that quercetin exhibits inhibitory potential against the phosphomonoesterase activity of PTPN22. As depicted in Figure 3a, quercetin displayed noteworthy inhibitory effects, particularly at concentrations of 750 µM and 1250 µM. Figure 3b shows that quercetin had a powerful inhibitory effect on PTPN22 with an IC_50_ of 29.59 µM. Ursolic acid, which served as a positive control, inhibited PTPN22 with an IC_50_ of 37.19 µM. Meanwhile, the double-reciprocal transformation plot, as shown in Figure 4, indicates a non-competitive mode of inhibition in the presence of 750 µM and 1250 µM quercetin. For a comprehensive understanding of the enzyme kinetics, Table 1 presents the Km and Vmax values for both 750 µM and 1250 µM, comparing conditions with and without quercetin. In the presence of 750 µM quercetin, Km is determined to be 11.11 µM, while Vmax is calculated as 0.209 µmol/min. In contrast, in the absence of quercetin, Km remains at 11.11 µM, but Vmax is notably higher at 0.598 µmol/min. Similarly, for 1250 µM quercetin, Km remains consistent at 11.11 µM, while Vmax registers at 0.251 µmol/min. Also, the Ki value of 550 µM was obtained for PTPN22 in the presence of 750 and 1250 µM (Figure 5). 

Values obtained are mean ± SEM of triplicates determination.

### 3.3. Docking Scores and Thermodynamic Information of Quercetin against PTPN22

Quercetin (−6.7 kcal/mol) had a lower negative docking score against PTPN22 compared to ursolic acid (−8.6 kcal/mol) (Table 2). An RMSD of 2.5 Å from the native inhibitor was obtained following optimal superimposition of docked quercetin and ursolic acid on the co-crystal structure of PTPN22. However, following 100 ns energy refinement, quercetin (−24.56 kcal/mol) had higher negative ΔG_bind_ relative to ursolic acid (−20.81) (Table 2).

**Figure 6:**
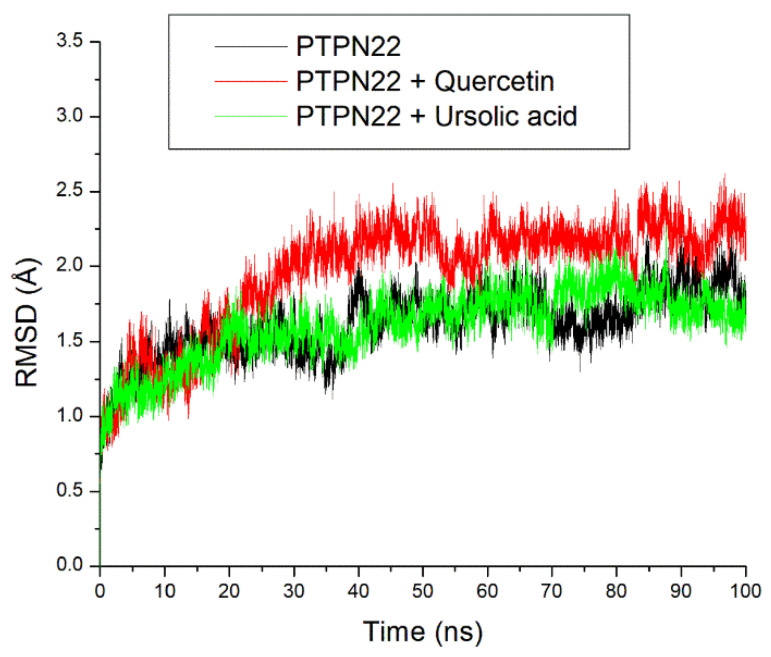
Relative root mean square deviation (RMSD) plots of alpha-carbon, quercetin, and ursolic acid against the PTPN22 over a 100 ns MD simulation period.

The RMSD plots revealed that the systems equilibrated before 10 ns while converging just before 60 ns (Figure 6). Quercetin + PTPN22 relatively had the highest fluctuation among the systems and consequently had the highest average RMSD at 2.02 Å. Ursolic acid + PTPN22 (1.60 Å) on the other hand had a lower average RMSD value than the apo-PTPN22 (1.68 Å) (Table 2). 

**Figure 7:**
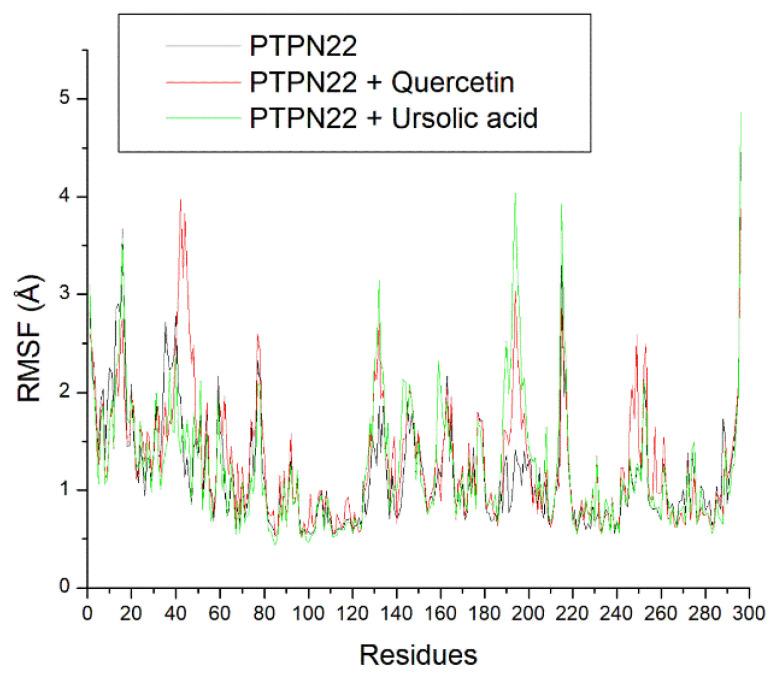
Relative root mean square fluctuation (RMSF) plots of alpha-carbon, quercetin, and ursolic acid against the PTPN22 over a 100 ns MD simulation period.

The RMSF plots showed lesser fluctuations of residues between 80 and 120, 220 and 240, and 260 and 280. Some of the active site residues such as Ala229, Ser228, Arg266, Gln278 were at the region of lesser fluctuation and on average (Figure 7), the bound systems had higher fluctuations of residues compared to the apo-PTPN22 (1.17 Å) with quercetin + PTPN22 having the highest fluctuations (1.29 Å) (Table 2).

**Figure 8:**
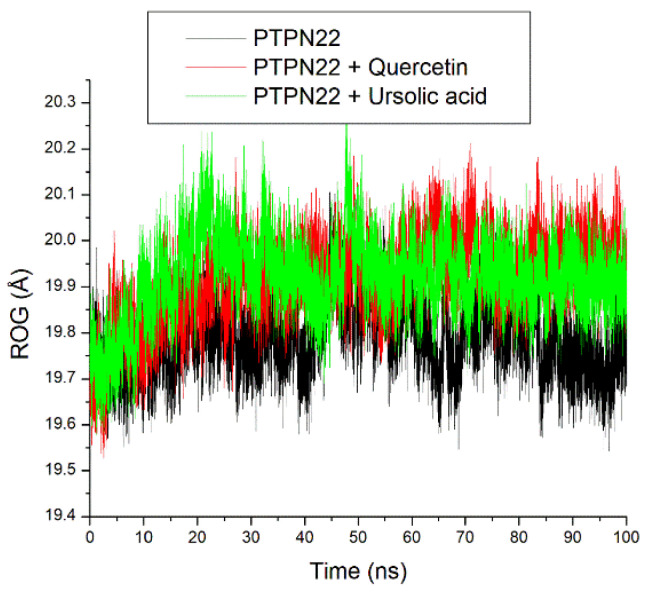
Relative radius of gyration (ROG) plots of alpha-carbon, quercetin, and ursolic acid against the PTPN22 over a 100 ns MD simulation period.

The ROG plots showed stable fluctuations of the systems just after equilibration at 10 ns (Figure 8). On average, small variance (0.15 Å) exits among the systems with quercetin + PTPN22 and ursolic acid + PTPN22 had relatively similar ROG values at 19.91 Å and 19.92 Å, respectively (Table 2). 

**Figure 9:**
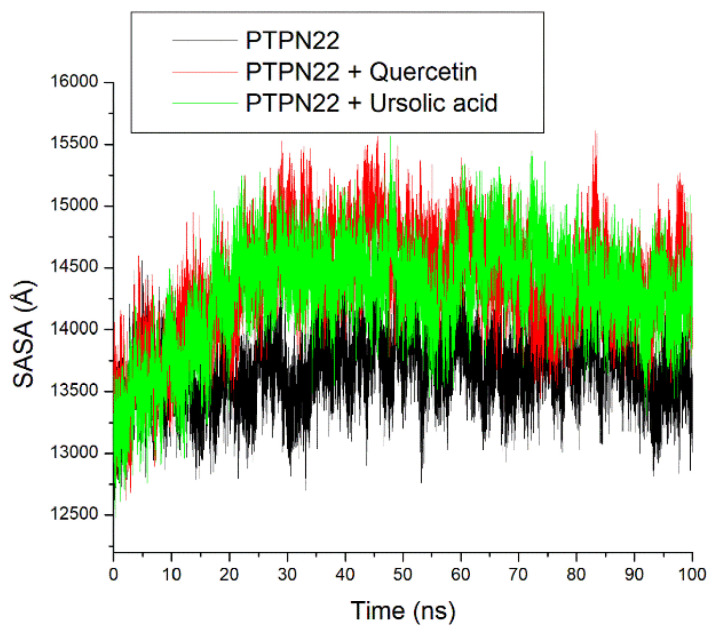
Relative solvent accessible surface area (SASA) plots of alpha-carbon, quercetin, and ursolic acid against the PTPN22 over a 100 ns MD simulation period.

Like the ROG plot, after equilibration at 10 ns, a stable fluctuation of SASA plots was observed for all the systems throughout the 100 ns simulation (Figure 9). A relatively comparable mean SASA value at 14,393.51 Å, 14,280.72 Å, and 14,229.35 exists for quercetin + PTPN22, ursolic acid + PTPN22, and apo-PTPN22, respectively (Table 2).

**Figure 10:**
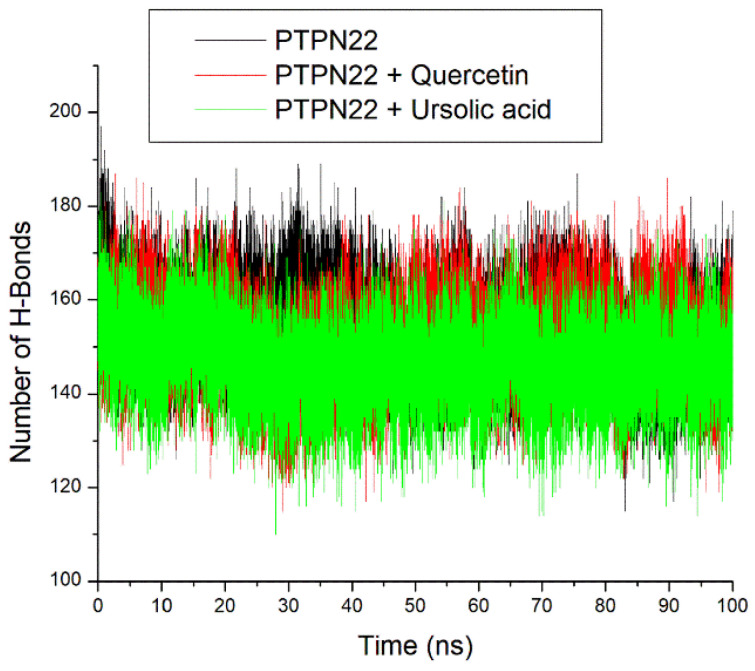
Relative number of intramolecular hydrogen bond plots of apo-PTPN22, quercetin-PTPN22, and ursolic acid + PTPN22 over a 100 ns MD simulation period.

The intramolecular hydrogen bond plots had stable fluctuations of systems (Figure 10). The apo-PTPN22 (151.05) had a marginally similar number of intramolecular hydrogen bonds compared to quercetin + PTPN22 and ursolic acid + PTPN22 at 151.72 and 151.71, respectively (Table 2).

**Figure 11:**
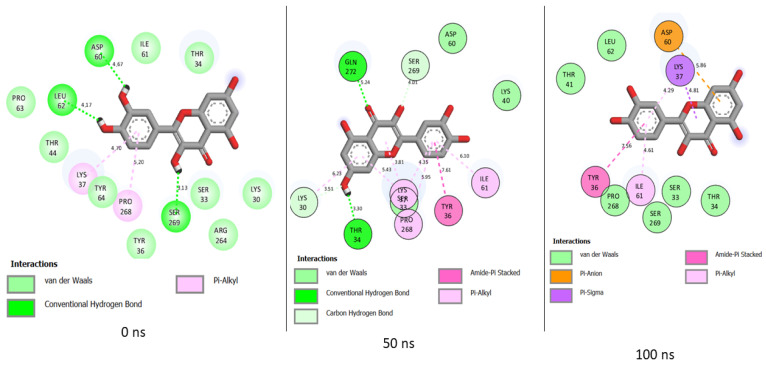
Plot of interactions of quercetin against PTPN22 at different time intervals during the 100 ns simulation.

**Figure 12:**
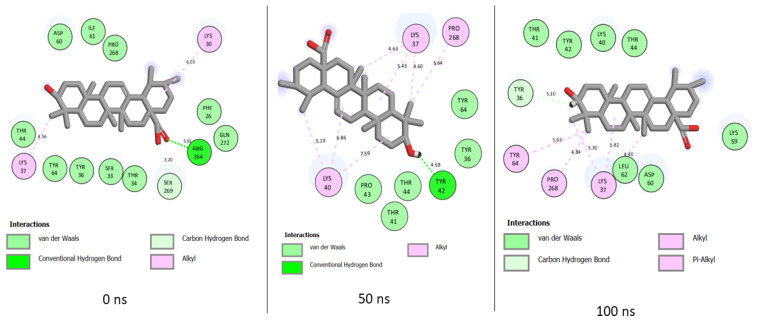
Plot of interactions of ursolic acid against PTPN22 at different time intervals during the 100 ns simulation.

The MD simulation of quercetin at the binding pocket of PTPN22 includes interactions such as van der Waals, hydrogen bond, Pi-Alkyl, Amide-Pi Stacked, Pi-Anion, and Pi-Sigma (Figure 11). On the other hand, only van der Waals, hydrogen bond, Alkyl, and Pi-Alkyl are involved with ursolic acid binding of PTPN22 during the MD simulation (Figure 12). While the nature of interaction in quercetin + PTPN22 plots increases as the simulation progresses, the total number of interactions reduces from 14 at 0 ns to 12 at 50 ns and 11 at 100 ns (Figure 11). Three hydrogen bonds were maintained in quercetin + PTPN22 at 0 ns and 50 ns, but none were included in the 100 ns interaction plots (Figure 11). Unlike quercetin + PTPN22 plots, the nature of interaction in ursolic acid + PTPN22 was consistent throughout the simulation (Figure 12). A reduced number of interactions from 14 to 13 to 12 at 0 ns, 50 ns and 100 ns was observed as ursolic acid + PTPN22 simulation progresses (Figure 12). However, several van der Waals interactions at 0 ns were replaced with Alkyl and Pi-Alkyl interactions as the simulation continued (Figure 12). While there were no consistent interactions in quercetin + PTPN22 plots, amino acid residues such as Ser269, Pro268, Asp60, Ile61, Ser33, Thr34, Tyr36 were constant at each of the time plots (Figure 11). However, in ursolic acid + PTPN22 plots, Alkyl interactions with Lys37 were consistent during the simulation. Also, amino acids such as Lys37, Pro268, Thr44, Tyr64 were consistent at each of the timeframes investigated (Figure 12). 

#### Quantum Information on Quercetin and Ursolic Acid

With a lower LUMO value of −2.09393 eV and a higher HOMO value at −6.07752, quercetin relatively had a lower energy gap (3.983582 eV), hardness (1.991791 eV), ionization energy (6.077517 eV), and chemical potential (−4.08573 eV) compared to ursolic acid (Table 3). Correspondingly, quercetin also had the highest softness (0.502061 eV), electron affinity (2.093934 eV), electronegativity (4.085726 eV), and electrophilicity index (4.190488 eV). 

## 4. Discussion

Since the mid-20th century, significant scientific advancements have transformed our understanding of cancer. According to the National Cancer Institute [4], these breakthroughs encompass early detection methods, specialized surgeries, radiation therapy, and chemotherapy drugs, all of which have revolutionized cancer treatment.

Recent research by Jassim et al. [7] points out the limitations of current therapies, emphasizing the need for novel strategies. Immunotherapy techniques like immune checkpoint blockade and adoptive T cell therapy, they suggest, offer advantages over traditional treatments such as chemotherapy, radiotherapy, and surgery.

Our study investigates the inhibition of PTPN22 by quercetin using both in vitro and in silico methods. PTPN22 is implicated in various autoimmune diseases, including cancer [20]. This work is consistent with the findings of other researchers [21,22] who have highlighted quercetin’s role in preventing cancer and oxidative stress, as well as its anti-inflammatory and antioxidant properties. Our evaluation of quercetin’s inhibitory activity on PTPN22 enzymatic activity yielded intriguing results. Quercetin exhibited non-competitive inhibition, consistent with its actions on other enzymes [23,24,25]. This pattern suggests that quercetin may have a similar inhibitory mechanism across different phosphatases.

The inhibition of PTPN22 by quercetin opens new possibilities for developing quercetin-based therapies for cancer and autoimmune diseases. This is especially pertinent given the well-documented association between PTPN22 polymorphisms and various autoimmune diseases [26]. Recent studies [6,27,28,29,30,31] have underscored PTPN22’s role in cancer immunotherapy and its potential as a therapeutic target.

Quercetin’s established anti-inflammatory and antioxidant properties complement its role in cancer prevention and treatment. It modulates signaling pathways, impacting apoptosis and autophagy, with inhibitory effects on cell proliferation and apoptosis observed in various cancer cell lines [32,33]. Furthermore, quercetin shows promise in addressing cardiovascular and neurodegenerative disorders [34,35,36,37]. These findings highlight quercetin’s multifaceted benefits and its potential significance in addressing cancer and immune-related diseases.

To further corroborate the in vitro findings, the ability of quercetin to efficiently bind PTPN22, a protein involved in the signaling that helps control the activity of the immune system cells (T cells), was carried out in silico. Targeting PTPN22 could afford the opportunity to augment cancer immunotherapy through at least two clinically validated classes of therapies and pathways—interferon alpha receptor (IFNAR) and T cell receptor (TCR) signaling [30]. Through a molecular docking study, it is possible to assess the geometric fitness of a compound when bound to a protein and the higher the negative docking score, the better the fitness and interaction of the molecule with the protein [38]. The lower negative docking scores of quercetins relative to ursolic acid against PTPN22 in this study specified better fitness of ursolic acid at the binding pocket of PTPN22. Findings from the docking validation denote a partial binding orientation between the docked compounds and the native inhibitor of PTPN22 suggesting the reliability of the docking study. However, as the molecular docking study is only a preliminary assessment of compound interactions with a target, further energy refinement and thermodynamic evaluation was carried out. Unlike the docking study, energy refinement highlights the advantage of quercetin relative to ursolic acid as an inhibitor of PTPN22. This observation is coherent with the report of Cerón-Carrasco [19] on the capability of virtual screening yielding inactive molecules. Thus, the further energy refinement as exploited in this study might increase the likelihood of quercetin finding usefulness as a potential PTPN22 inhibitor at the preclinical and clinical phase of drug development. 

The root mean square deviation (RMSD) quantifies the time-dependent variance of a complex structure from its apo structure, where reduced RMSD values signify enhanced stability of the complex. The equilibration and convergence of the systems during the 100 ns MD simulation denote the efficiency and accuracy of the simulation, suggesting its reproducibility [39]. The relatively lower RMSD value of ursolic acid + PTPN22 compared to quercetin + PTPN22 and the apo-PTPN22 suggest its better structural stability and thus an advantage for ursolic acid as a PTPN22 inhibitor. Worth noting however is that quercetin + PTPN22 also had an RMSD value that could encourage protein inactivation [38]. The mobility of PTPN22′s residues was examined by analyzing the RMSF. The RMSF value considers the average volatility of atoms and residues in a protein structure over a simulation time, which can be linked to their capacity to form intra- and intermolecular stable bonds. The observation of some PTPN22 active site residues such as Ala229, Ser228, Arg266, Gln278 at the region of lesser fluctuation might mean strong intermolecular binding during the simulation. This observation is further substantiated by the average RMSF value for the bounded systems that were <3 Å limit [38]. However, quercetin + PTPN22 having a higher RMSF value than ursolic acid + PTPN22 might suggest the better advantage of ursolic acid as a PTPN22 inhibitor and is coherent with the RMSD findings of this study. The ROG estimates the extent of compactness and folding of a complex during a simulation [40]. High compactness and folding suggest a better degree of thermodynamic orderliness and sometimes stability in a complex. Stable fluctuations of ROG plots for all the systems after equilibration might mean that quercetin and ursolic acid do not instigate thermodynamic disorderliness in PTPN22 after binding. This is further highlighted as small variance exits between the bounded systems and apo-PTPN22, suggesting the thermodynamic compatibility of quercetin and ursolic acid as PTPN22 inhibitors. Like the ROG, a stable fluctuation of SASA plots and number of intramolecular hydrogen bond plots for all the systems after equilibration might indicate that quercetin and ursolic acid are thermodynamically suited as PTPN22 inhibitors. This observation is further corroborated as a marginally comparable mean SASA, and the number intramolecular hydrogen bonds values exist between apo-PTPN22 and the bound systems. 

Factors relating to numbers, nature, and distance of interactions with critical amino acids of a protein influence the ability of a ligand to bind and inactivate a protein [39]. In quercetin + PTPN22 interaction plots, more important bonds such as Pi-Alkyl, Amide-Pi Stacked, Pi-Anion, and Pi-Sigma replaced several van daal Waals interactions that were found at the early stage of the simulation. The involvement of quality interaction as the simulation progresses might have lowered the effects of the continuous reduction in the total number of interactions in quercetin + PTPN22 complex. Ursolic acid + PTPN22 also had several van der Waals interactions at the beginning of the simulation replaced by Alkyl and Pi-Alkyl interactions excluding Amide-Pi Stacked, Pi-Anion, and Pi-Sigma that were found in quercetin + PTPN22 interaction plots. The importance of Amide-Pi Stacked, Pi-Anion, and Pi-Sigma bonds in drug discovery have been highlighted in a previous study [41], thus their presence in quercetin + PTPN22 plots might have contributed to the higher binding free energy in the complex relative to ursolic acid + PTPN22. Moreover, as quercetin + PTPN22 and ursolic acid + PTPN22 had a comparable number of interactions during the simulation. The findings that no consistent interactions were observed in quercetin + PTPN22 with only one in ursolic acid + PTPN22 during the simulation might be due to the replacement of van der Waals interactions with stronger bonds as the simulation proceeded. This observation might be a benefit for ursolic acid and quercetin as inhibitors of PTPN22. Conserved amino acid residues such as Ser269, Pro268, Asp60, Ile61, Ser33, Thr34, Tyr36 and Lys37, Pro268, Thr44, Tyr64 in quercetin + PTPN22 and ursolic acid + PTPN22, respectively, were observed to be important in the binding of the ligands during the simulation. 

The energy gap between the LUMO and the HOMO parameters of a compound provides insight into their stability, with a higher energy gap suggesting high stability. Higher stability could deter the binding of a compound to a protein, and vice versa for a smaller energy gap [16,17]. The lower energy gap of quercetin relative to ursolic acid might mean quercetin is less stable and thus it has a better chance of binding PTPN22. Chemical hardness and softness also provide relevant information about the reactivity of a molecule, and based on Pearson’s HSAB principle 21, higher chemical softness and lower chemical hardness mean higher reactivity of the compound [18]. Like the energy gap, the higher softness of quercetin means higher reactivity relative to ursolic acid. This is further corroborated by the lower electron affinity, electronegativity, and electrophilicity index of quercetin relative to ursolic acid, which might indicate quercetin had better capability to attract and donate electrons at the active site of PTPN22 [42]. The lesser stability, higher reactivity, and enhanced capability to attract and donate electrons of quercetin might have impacted its higher binding free energy against PTPN2 relative to ursolic acid in this study. 

While the present study provides valuable insights into quercetin’s inhibitory activity on PTPN22, further studies are necessary to fully explore its potential. In vivo studies and clinical trials would be essential to evaluate its efficacy and safety in real-world applications. Additionally, investigations into the specific mechanisms of quercetin’s interaction with PTPN22 and its effects on immune responses should be undertaken to gain a comprehensive understanding of its therapeutic potential. 

## 5. Conclusions

In conclusion, this study successfully evaluated quercetin and demonstrated its non-competitive inhibitory potency on PTPN22 enzymatic activity. These findings are consistent with previous research on quercetin’s presence in plant sources and its non-competitive inhibition of various enzymes. Inhibiting PTPN22 represents a promising cancer immunotherapeutic strategy, and our research offers proof of concept for the potential translatability of this target using a lead compound. Given PTPN22’s role in regulating T cells and macrophages in tumor defense, we propose that PTPN22 inhibitors could emerge as a unique form of cancer immunotherapy with broad immunomodulatory effects, especially when combined with other immunotherapeutic strategies. Further exploration in this area could lead to innovative and effective treatments for cancer and immune-related disorders.

## Figures and Tables

**Table 1:** Kinetics of inhibition parameters for PTPN22 inhibition by quercetin.

Compound/Concentration	Km (µM)	Vmax (µmol/min)
0	11.11	0.598
750	11.11	0.209
1250	11.11	0.251

**Table 2:** Docking scores, binding free energy, and thermodynamic data of quercetin against PTPN22.

Complex	Docking Scores (Kcal/mol)	ΔG_bind_ (Kcal/mol)	RMSD (Å)	RMSF (Å)	ROG (Å)	SASA (Å)	H-Bonds
Apo			1.68 ± 0.2	1.17 ± 0.5	19.77 ± 0.07	14229.35 ± 290	151.05 ± 8.57
Quercetin	−6.7	−24.56	2.02 ± 0.3	1.29 ± 0.6	19.91 ± 0.08	14393.51 ± 389	151.72 ± 8.48
Ursolic acid	−8.6	−20.81	1.60 ± 0.2	1.24 ± 0.6	19.92 ± 0.08	14280.72 ± 405	151.71 ± 8.26

**Table 3:** The CDFT of quercetin and ursolic acid using DFT calculated by B3LYP/6-31G + (dp).

CDFT Descriptors (eV)	Quercetin	Ursolic Acid
E_LUMO	−2.09393	−0.28403
E_HOMO	−6.07752	−6.31239
Energy gap (Δ*E*)	3.983582	6.028357
Ionization energy (*I*)	6.077517	6.312391
Electron affinity (*A*)	2.093934	0.284034
Hardness (*η*)	1.991791	3.014179
Softness (*S*)	0.502061	0.331765
Electronegativity (*χ*)	4.085726	3.298213
Chemical potential (*μ*)	−4.08573	−3.29821
Electrophilicity index	4.190488	1.804506

## Data Availability

Data presented in this study are available on request from the corresponding author.
